# When does recurrent connectivity improve neural population coding?

**DOI:** 10.1186/1471-2202-15-S1-P49

**Published:** 2014-07-21

**Authors:** Joel Zylberberg, Eric Shea-Brown

**Affiliations:** 1 Department of Applied Mathematics, University of Washington, Seattle, WA 98195, USA; 2 Department of Physiology and Biophysics, University of Washington, Seattle, WA 98195, USA

## 

Neural systems contain many cells, and an important problem is to understand if and how those neurons work together to form a functioning system. In sensory neuroscience -- which is our focus -- this function is to encode information about a stimulus so that it can be transmitted to other brain areas. An experimentally accessible, and hence popular, way to assess collective behavior is to measure the trial-to-trial covariability in the responses of multiple neurons over repeats of the same stimulus, called noise correlations. Repeat presentations of the same stimulus yield different responses on each trial, and that variability is typically correlated across different neurons [1,2] (although, see [3] for one counterexample).

In the past two decades, the impact of these noise correlations on population coding has generated great interest. While nothing can add more information about the stimulus than was contained in the inputs (the data processing inequality), noise correlations determine the extent to which noise in the neural system degrades the amount of information that a neural population conveys about a stimulus (for example, see [4-6]).

There are two main ways in which these noise correlations can be generated: the cells may receive common (noisy) input from (some of) the same upstream source(s), or the cells may be (recurrently) coupled to one another. At the same time, most work on noise correlations and population coding (with a few notable exceptions, including [7,8]) ignores their mechanistic origins. Interestingly, the few studies that have considered the mechanistic origins of noise correlations [7,8] have concluded that recurrent connectivity tends to hinder population coding, or has little overall effect: even though recurrent coupling can “sharpen” neural tuning curves, this advantage is more than offset by the fact that it generates harmful noise correlations. This begs the question of when -- if ever -- recurrent coupling can improve population coding, and of whether noise correlations with different mechanistic origins have different impacts on coding performance?

To address these issues, we are investigating models in which groups of cells with, and without, recurrent coupling are driven by noisy inputs. The cells themselves are then noisy spike generators, and we are varying the fraction of shared inputs to the cells (which modifies the noise correlations due to common input), and the inter-neuronal connectivity, and computing the coding capacity of the resultant networks. Preliminary results indicate that, in some cases – similar to those discussed in [9] – recurrent coupling can combat the cell-intrinsic variabililty enough that the population’s coding performance is improved even though the noise correlations the are generated by that coupling do themselves hinder that coding performance. We are in the process of expanding and generalizing on this work.

Support for this work came from NSF grants DMS-1122106, and CRCNS DMS-1208027 and a Burroughs- Wellcome Fund Career Award at the Scientific Interface to ESB.
